# Myocardial Autophagy after Severe Burn in Rats

**DOI:** 10.1371/journal.pone.0039488

**Published:** 2012-06-29

**Authors:** Rong Xiao, Miao Teng, Qiong Zhang, Xiao-hua Shi, Yue-sheng Huang

**Affiliations:** Institute of Burn Research, State Key Laboratory of Trauma, Burns and Combined Injury, Southwest Hospital, The Third Military Medical University, Chongqing, China; Kaohsiung Chang Gung Memorial Hospital, Taiwan

## Abstract

**Background:**

Autophagy plays a major role in myocardial ischemia and hypoxia injury. The present study investigated the effects of autophagy on cardiac dysfunction in rats after severe burn.

**Methods:**

Protein expression of the autophagy markers LC3 and Beclin 1 were determined at 0, 1, 3, 6, and 12 h post-burn in Sprague Dawley rats subjected to 30% total body surface area 3rd degree burns. Autophagic, apoptotic, and oncotic cell death were evaluated in the myocardium at each time point by immunofluorescence. Changes of cardiac function were measured in a Langendorff model of isolated heart at 6 h post-burn, and the autophagic response was measured following activation by Rapamycin and inhibition by 3-methyladenine (3-MA). The angiotensin converting enzyme inhibitor enalaprilat, the angiotensin receptor I blocker losartan, and the reactive oxygen species inhibitor diphenylene iodonium (DPI) were also applied to the *ex vivo* heart model to examine the roles of these factors in post-burn cardiac function.

**Results:**

Autophagic cell death was first observed in the myocardium at 3 h post-burn, occurring in 0.008 ± 0.001% of total cardiomyocytes, and continued to increase to a level of 0.022 ± 0.005% by 12 h post-burn. No autophagic cell death was observed in control hearts. Compared with apoptosis, autophagic cell death occurred earlier and in larger quantities. Rapamycin enhanced autophagy and decreased cardiac function in isolated hearts 6 h post-burn, while 3-MA exerted the opposite response. Enalaprilat, losartan, and DPI all inhibited autophagy and enhanced heart function.

**Conclusion:**

Myocardial autophagy is enhanced in severe burns and autophagic cell death occurred early at 3 h post-burn, which may contribute to post-burn cardiac dysfunction. Angiotensin II and reactive oxygen species may play important roles in this process by regulating cell signaling transduction.

## Introduction

Autophagy plays a major role in the physiological cellular degradation and recycling of long-lived proteins and organelles, maintaining cellular homeostasis and adaptation to nutrient depletion [1]. Autophagy represents a programmed and dynamic process that proceeds by sequestration of cellular material into double membrane vacuoles that dock to and fuse with lysosomes to form autophagic vacuoles (i.e. autophagosome), where the contents are degraded by lysosomal hydrolases and the resulting macromolecules are recycled [2]. During the assembly of the autophagosome, microtubule associated protein 1 light chain 3 (LC3), originally synthesized as a precursor cytosolic protein, is converted from LC3 I to LC3 II. The ratio of LC3 II to LC3 I, therefore, is an established indicator of autophagy [3]. Autophagy occurs at basal levels, but can also be induced by stress conditions such as hypoxia or ischemia/reperfusion [4], [5]. Autophagy not only serves as a cell survival mechanism, but may also lead to cell death (type II programmed cell death) [6]. Autophagic cell death contributes during organ development and in neurogenerative disorders such as Parkinson’s disease [7]. The ubiquitin/protein degradation system is associated with the autophagic machinery [8], therefore ubiquitin expression in tissues can be used to determine autophagic cell death [9].

The functional role of autophagy during cardiac ischemia/reperfusion is controversial. Autophagy was shown in one report to inhibit apoptosis in chronically ischemic pig myocardium, serving as an injury response mechanism [10]. Autophagy was also found to be protective in HL-1 myocytes subjected to simulated ischemia/reperfusion [11], [12]. However, other reports suggest that autophagy plays a detrimental role in myocardial ischemia/reperfusion injury. Inhibition of autophagy by 3-methyladenine (3-MA) prevents H9c2 cell death during glucose deprivation [13]. Further, Beclin 1-mediated autophagic cell death can be protectively inhibited by urocortin in cardiac myocytes under ischemia/reperfusion [14]. Matsui et al. have proposed that autophagy may be protective during ischemia while detrimental during reperfusion, and the differential effects are dependent on distinct signal pathways [15]. Therefore, the exact roles of autophagy in cardiac ischemia/reperfusion remain unclear. More information on the time course for the induction and progression of autophagy in different stress conditions in the heart is needed.

In severe burns, the myocardium undergoes both hypoxic and ischemic injury. Our previous reports have shown that serum cardiac troponin I, a specific myocardial structural protein as an indicator of injury, increases 1 h post-burn, accompanied by decreased heart function which still does not recover at 24 h post-burn [16]. Under the stress of severe burns, the blood flow to the heart declines very early [17], which may result from the instant activation of the cardiac renin-angiotensin system (RAS) [18]. It has been reported that indicators of apoptosis, such as caspase-3 activity, increase at 3 h after severe burns, and the significant morphologic alterations characteristic of apoptosis can be found at 6 h post-burn [19]. At 6 h post-burn, few of the myocardial fibers are breaking and dissolving, a sign of cardiomyocyte oncosis [16]. However, in the setting of cardiac ischemia induced by burn injury, little is known about the roles that autophagy plays. Whether autophagy is a protective mechanism or a cause for early cardiac cell death in severe burn and how autophagy affects cardiac function is unclear. The present study is the first report to shed light on the roles of autophagy in cardiac damage after severe burns.

## Results

### Changes of Autophagy Marker Proteins

**Figure 1:**
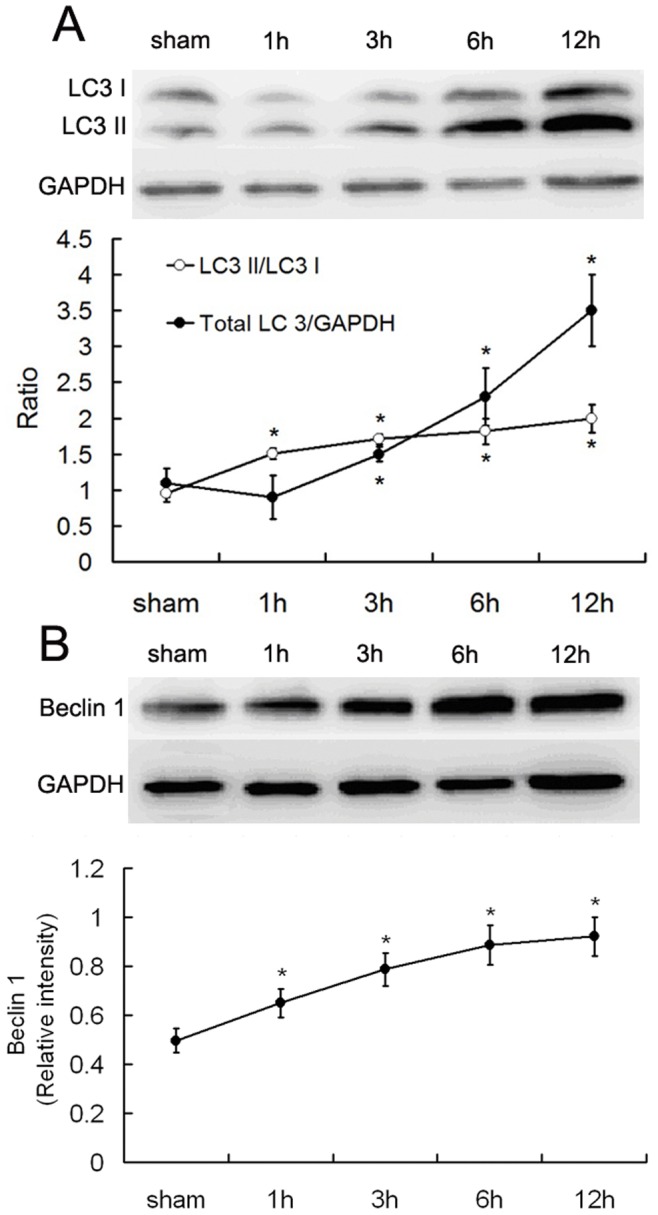
Myocardial LC3 and Beclin 1 expression following severe burns in rats. Immunoblotting was performed on proteins extracted from myocardial tissue of rats exposed to 3^rd^ degree burns over 30% of the total body surface area or sham controls. **A.** As an indicator of autophagy, LC3 II/LC3 I ratio rose from 1 h to 12 h post-burn. The total level of LC3 protein also increased, beginning at 3 h and continuing to rise through 6 and 12 h post-burn. The sample size was n = 6 per group for sham and each of the time points, * p<0.05 vs sham. **B.** Beclin 1 expression was robustly expressed at 1 h post-burn and continued to follow an upward tendency. The sample size was n = 5 for each group, * p<0.05 vs sham.

To determine whether cardiac autophagy was enhanced under severe burn conditions, we examined for LC3 and beclin 1 expression. LC3 II, which is localized in autophagosome membranes, is converted from LC3 I during processing [3]. The half-life of autophagosomes is very short, usually only a few minutes. The LC3 II/ LC3 I ratio, therefore, can sensitively reflect the current autophagic status. Our results showed that the LC3 II/ LC3 I ratio significantly rose at 1 h post-burn in the myocardium, and the total level of LC3 proteins also increased later at 3h post-burn. Both of them continued to increase through the 12 h time point (Fig. 1A).

Beclin-1 is also known as an autophagy-related protein involved in the initiation of autophagasome formation. As reported, Beclin 1-mediated signal transduction may lead to autophagic cell death [14], and the expression of Beclin 1 is enhanced during the reperfusion stage, but not during ischemia [5]. Our results showed that, similar to LC3 II/ LC3 I, beclin 1 expression increased robustly from 1 h to 12 h post-burn (Fig. 1B).

### Changes in Cardiac Function

**Figure 2:**
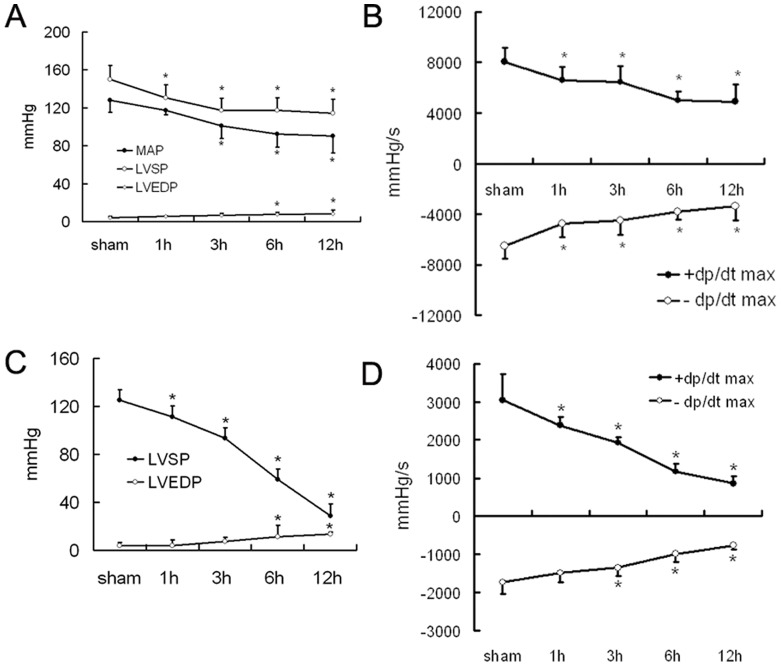
Post-burn cardiac function *in vivo* and *ex vivo* in a Langendorff preparation. Rats subjected to 3rd degree burns affecting 30% of total body surface area were established as a burn model. **A.** These cardiac mechanical parameters were obtained with an intubation method from the carotid artery of the rats under anesthesia. The left ventricular systolic pressure (LVSP) and mean arterial pressure (MAP) decreased from 1 h to 3 h post-burn and continued to decline in the following time points. The left ventricular end-diastolic pressure (LVEDP) rose from 6 h to 12 h post-burn. The sample size was n = 8 for each group, * p<0.05 vs sham. **B.** Reflecting cardiac contraction and relaxation, the maximal rates of left ventricular systolic pressure increase and diastolic pressure decrease (i.e., +dp/dt max and -dp/dt max, respectively) fell from 1 h to 12 h post-burn. The samples size was n = 8 for each group, * p<0.05 vs sham. **C and D.** In isolated hearts perfused with a Langendorff apparatus, the recorded cardiac mechanical parameters LVSP and ± dp/dt max decreased from 1 h to 12 h post-burn, similar to the changes seen *in vivo*. The sample size was n = 5 for each group, * p<0.05 vs sham.

We determined cardiac function after severe burn *in vivo* and in isolated hearts. The *in vivo* myocardial mechanical parameters, especially the maximal rate of the rise/drop of left ventricular pressure (±dp/dt max), indicated that cardiac contraction and relaxation were impaired very early, at 1 h post-burn, and further declined by 12 h post-burn (Fig. 2A, B). In the *ex vivo* perfused hearts isolated from 1 h, 3 h, 6 h, and 12 h following burn, cardiac function was also depressed (Fig. 2C, D).

### Autophagic, Apoptotic, and Oncotic Cell Death

**Figure 3:**
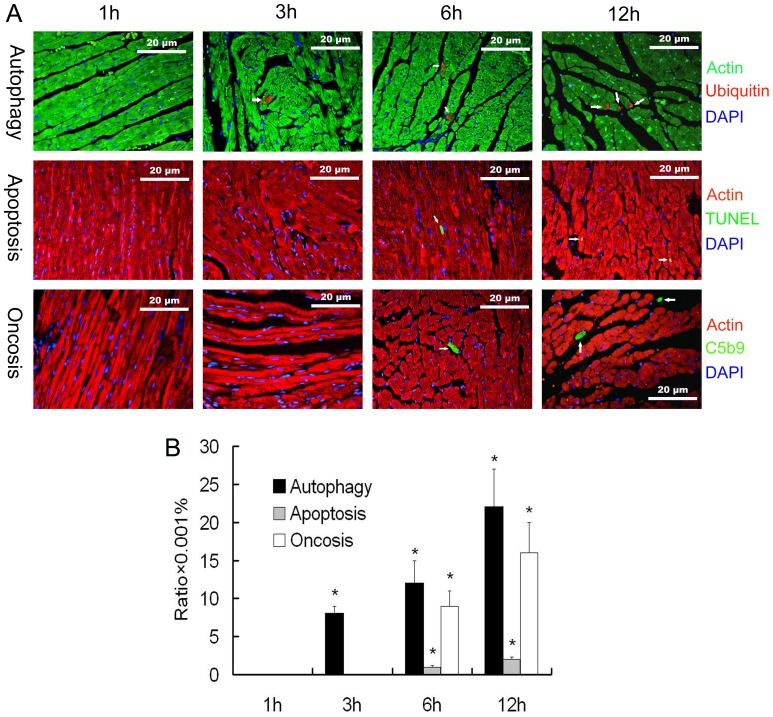
Myocardial immunofluorescence of autophagic, apoptotic, and oncotic cell death. A. Representative immunofluorescence images of myocardium with different staining targets in rats with 3rd degree burns over 30% of total body suface area. As indicated by the white arrows, cardiomyocytes with ubiquitin accumulation were mostly loss of nuclei and occupied partially by the positive staining. The TUNEL staining co-localized with DAPI staining in nuclei. The complement membrane attack complex C5b9 staining frequently appeared in the myocyte periphery and showed progression toward the cell center. **B.** Quantification of autophagic (ubiquitin positive), apoptotic (TUNEL positive), and oncotic (C5b9 positive) cardiomycyte death showed that autophagic cell death could be found at 3 h post-burn, while the other two types of cell death occurred later. All three types of cell death increased further at 6 and 12 h post-burn. The sample size was n = 8 for each group, * p<0.05 vs the 1 h group.

The visualization of autophagosomes and increased ubiquitin conjugation in dying cells indicate that autophagy is a non-apoptotic form of programmed cell death [9]. C5b9, the complement membrane attacking complex, provided a tool for detection of early oncosis [20]. The immunofluorescence results showed that no cell death was seen in the myocardium at 1 h post-burn, while at 3 h after severe burns, autophagic cell death could be found morphologically at a ratio of 0.008 ± 0.001%, without apoptosis and oncosis. From 6 h to 12 h post-burn, 3 types of cell death presented together, and all increased in quantities with duration of injury (Fig. 3A, B).

### Influences of Autophagy Regulation on Cardiac Function

**Figure 4:**
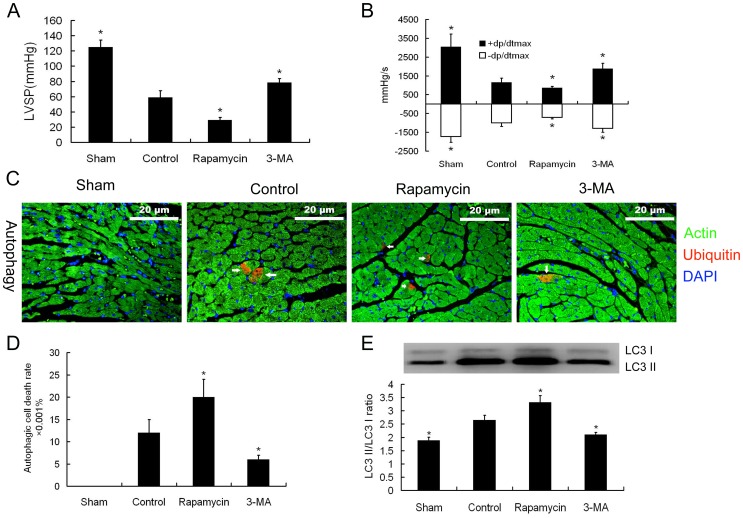
Effects of autophagic regulation on cardiac function. The hearts were isolated from rats with 3^rd^ degree burns over 30% of total body surface area at 6 h post-burn. Continuous K-H buffer perfusion without medication was used as a negative control. **A and B.** The left ventricular systolic pressure (LVSP) and the maximal rate of the rise/drop of left ventricular pressure (± dp/dt max) decreased in the hearts perfused with the autophagy activator rapamycin. While the ± dp/dt max increased with treatment of the autophagy inhibitor, 3-methyladenine (3-MA). The sample size was n = 5 for each group, * p<0.05 vs control. **C.** Representative immunofluorescence images of myocardial autophagic cell death (ubiquitin positive, red) in isolated hearts perfused with rapamycin, 3-MA, or non-medication (control). **D.** Quantitation of autophagic cell death showed that autophagy increased in the rapamycin treated group and decreased in the 3-MA treated group. The sample size was n = 5 for each group, * p<0.05 vs control. **E**. Immunoblotting results confirmed the activation or inhibition effects on autophagy by rapamycin and 3-MA, respectively. The sample size was n = 5 for each group, * p<0.05 vs control.

We tested if activation or inhibition of autophagy affects cardiac function in rats suffering from severe burn. As shown in Fig. 3, hearts from 6 h post-burn had an intense autophagic response. The 6 h time point, therefore, was chosen for evaluation in the isolated heart perfusion models. Our results showed that the activation of autophagy by rapamycin caused a decrease in cardiac function, while inhibition of autophagy by 3-MA resulted in improved cardiac function (Fig. 4A, B). The effects on autophagic cell death in the myocardium of rapamycin and 3-MA treated hearts were confirmed by immunofluorescence staining (Fig. 4C, D). Immunoblotting for LC3 II/ LC3 I ratio also demonstrated that rapamycin activates autophagy, while 3-MA inhibited autophagy (Fig. 4E).

### Effects of Enalaprilat, Losartan, and DPI on Autophagy and Cardiac Function

In order to explore the mechanisms of autophagic cardiomycyte death in severe burn rats, we evaluated whether the local cardiac RAS, which is activated immediately following severe burns and contributes to cardiac dysfunction [18], was involved in this model. We inhibited the generation of Ang II by enalaprilat and blocked the AT1 receptor by losartan to determine the changes in autophagy and cardiac function. We also examined the association between ROS and autophagy in severe burns by administering diphenylene iodonium (DPI), an inhibitor of the ROS-generating enzymes NADPH oxidase and nitric oxide synthase.

**Figure 5:**
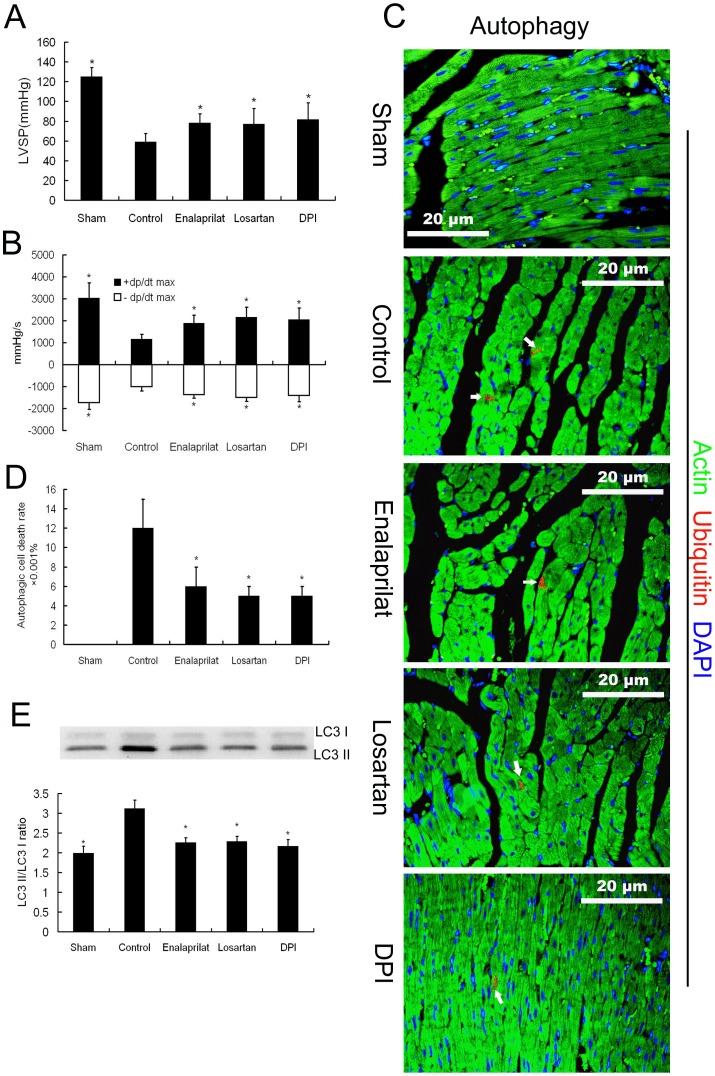
Effect of enalaprilat, losartan, or DPI on autophagy and cardiac function. The hearts were isolated from rats subjected to 3^rd^ degree burns over 30% of total body surface area at 6 h post-burn. Continuous K-H buffer perfusion without medication was used as a negative control. **A and B.** The left ventricular systolic pressure (LVSP) and the maximal rate of the rise/drop of left ventricular pressure (± dp/dt max) increased in isolated hearts perfused with enalaprilat, losartan, or DPI. The sample size was n = 5 for each group, * p<0.05 vs control. **C.** Representative immunofluorescence images of myocardial autophagic cell death (ubiquitin positive, red) in isolated hearts perfused with enalaprilat, losartan, DPI or non-medication (control). **D.** Quantitation of autophagic cell death showed that it decreased in the hearts medicated with enalaprilat, losartan, or DPI. The sample size was n = 5 for each group, * p<0.05 vs control. **E.** Immunoblotting results confirmed the inhibitory effects on autophagy by enalaprilat, losartan, or DPI treatment. The sample size was n = 5 for each group, * p<0.05 vs control.

The results showed that inhibition of Ang II, blockage of AT1 receptor, or inhibition of ROS production, improved cardiac function in isolated 6 h post-burn hearts (Fig. 5A, B). Compared with the control group, the hearts of the treated groups underwent less autophagic cell death (Fig. 5C, D). The immunoblotting results confirmed the inhibitory effects of these reagents on myocardial autophagy in severe burns (Fig. 5E).

## Discussion

In the very early stage of myocardial response to severe burns, leakage of myocardial-specific structural proteins and histopathologic changes occur and cause cardiac pumping deficit and decreased circulating blood volume, which in turn is an important initiating factor for other organ hypoxia injuries such as liver, kidney, and intestine [16]. In severe burn patients, multiple organ dysfunction syndrome (MODS) has been reported to occur at a 28.1% incidence and is very difficult to reverse [21]. In addition, MODS is also associated with a high mortality rate, ranging from 78%–98% among studies. Although the prophylactic treatment of MODS has improved survival rates in recent years, MODS remains a highly relevant clinical complication in severe burn patients [22]. As a result, our research team has been engaged in searching for the key issues linked to the occurrence of MODS in severe burns. Since cardiac dysfunction is an initiating factor in the pathological response of MODS, we focused on the effects of burn on myocardial injury. Our previous reports showed that apoptosis [19] and damage to mitochondria and cytoskeleton [23], [24] were major mechanisms for the myocardial injuries. However, these mechanisms do not totally explain the prompt occurrence of cardiac injury and dysfunction after severe burns. Cardiomyocyte loss has been suggested to be an important causative factor for the decline in cardiac function [25]. Severe burn-induced cardiac damage and dysfunction partially results from cardiomyocyte loss due to apoptosis [19]. Since autophagic cell death is an independent mode of cell death distinct from apoptosis and oncosis, it is important to measure autophagy in the setting of burn injury [26]. Therefore, we designed these experiments to investigate the roles of another type of programmed cell death, autophagy, in myocardial injuries during early stages of severe burns.

As shown in our results, autophagy was enhanced in myocardium at 1 h post-burn and gradually increased over the 12 h time course. In the process of autophagy, with the amount of autophagosome increasing, not only is LC3 I continuously converted to LC3 II (i.e., LC3 II /LC3 I ratio rises), but the total level of LC3 protein is also upregulated. The precursor of LC3 protein is constantly synthesized [27], [28]. Our results also showed that the LC3 II /LC3 I ratio rose, accompanied by the increase of the total LC3 protein level. However, autophagy plays dual roles in myocardial ischemia and hypoxia injuries. Autophagy serves as a protective mechanism under moderate hypoxia, while it causes cell death under critical hypoxia [4]. Some reports showed that the protective and detrimental functions of autophagy depend on different cell signaling pathways, and autophagy plays opposite roles in myocardial ischemia and reperfusion [5], [15], [29]. Taken together, autophagy can induce survival or death depending on the extent of stress. Although autophagy was enhanced 1 h post-burn, autophagic cell death was not found histologically until 3 h post-burn, when the quantity of autophagic cell death gradually increased in the following time points. This result indicates that autophagy may be protective within 3 h after severe burns, when the compensation mechanisms of organs can accomodate a quite moderate hypoxia condition in myocardium. After 3 h post-burn, in the setting of continuous hypoxia and possible effects of reperfusion, autophagy becomes detrimental and stimulates cardiomyocyte death.

Our previous work has demonstrated that apoptosis plays an important role in cardiac damage of the severely burned rats [19]. The issue of apoptotic cell death in myocardium after severe burns is not yet entirely clear, because values for apoptotic rates vary from very high [19] to almost absent [30]. However, in accordance with our results, these reports did not find any substantial levels of myocardial apoptosis within 6 h post-burn. Many reports show that apoptosis is quite limited relative to autophagy or oncosis in failing hearts [9], [31]. Narula’s concept of interrupted apoptosis is of special interest in this context and might explain the low apoptotic rate observed in the present study [32]. Because of the lack of knowledge of the time needed to complete the DNA fragmentation process and to remove apoptotic cells from the tissue, the calculated rate of apoptosis may be higher than actual. The present results show that autophagy preceded apoptosis, and the extent of autophagic cell death exceeded apoptosis rates. Since the proportion of autophagy is a greater contributor in cardiomyocyte loss in severe burns than apoptosis, autophagic cell death may contribute significantly to cardiac function impairment in severe burn.

We performed a comparative study of autophagy on perfused hearts isolated from rats of 6 h post-burn by rapamycin activation and 3-MA inhibition of autophagy. Under enhanced autophagy, the quantity of autophagic cell death increased, accompanied by cardiac deficits. Under autophagy inhibition, the amount of autophagic cell death decreased and cardiac function was enhanced. This suggests that in the pathological process of severe burn, autophagy in the myocardium mainly plays a detrimental role by directly inducing cardiac dysfunction. The significance of our present findings is demonstrating that control of autophagy occurs during a certain time course, i.e. after 3 h post-burn, which may enhance cardiac pumping abilities and reverse the dysfunction, suggesting that this is a reasonable medical intervention target point to prevent MODS.

Further studies on how autophagy is triggered in severe burns and how it causes cardiomycyte death are warranted. To provide mechanistic insight, we added three kinds of inhibition agents (ACEI, AT1 receptor blocker, and an inhibitor of ROS production) to the perfusion fluid to the isolated hearts at 6 h post-burn. All three agents inhibited autophagy and enhanced cardiac function. Porrello et al [33], [34] recently reported that autophagy is enhanced by Ang II through an activating AT1 receptor approach. As reported, the cardiac local RAS is activated quickly after severe burns and the concentration of Ang II increases in the myocardium [18]. According to these studies, the accumulation of Ang II activates cardiac autophagy. In concurrence with this idea, inhibition of Ang II generation or blockage of its effective receptor remarkably mitigated autophagy and its negative effects on cardiac function. It has been reported that ROS can activate autophagy by damage to mitochondria and regulate autophagy in protective or fatal effects [35]. Our results also showed that DPI could alleviate autophagy by inhibition of ROS in isolated perfused hearts at 6 h post-burn. This indicates that ROS may also play a crucial role in the cell signaling of autophagic cell death.

### Conclusion

Myocardial autophagy is significantly enhanced after severe burns, and autophagic cell death occurs earlier than apoptosis and oncosis in the myocardium. The reduction in post-burn cardiac function occurred in parallel to increased autophagic cell death, and regulating the extent of autophagy altered cardiac function in isolated perfusion models. Our results suggest that autophagic cell death contributes significantly to post-burn cardiac dysfunction. In addition, Ang II and ROS play important roles in the autophagic cell death signal transduction pathways.

## Materials and Methods

This study conforms to the NIH Guide for the Care and Use of Laboratory Animals (NIH publication no. 85–23, revised 1996).

### Experimental Animals

Healthy adult male Sprague-Dawley rats (200 g–250 g; n = 120) were used in the study. The animal experiments were conducted according to protocols reviewed and approved by the Animal Experimental Ethics Committee of the Third Military Medical University, Chongqing, China (Permit Number SYXK-CQ-20070002). Animals were allowed 5 days to acclimate to their surroundings. Food and water for rats were available ad libitum throughout the experimental protocol.

### Groups and Burn Procedure

Rats were randomized into burn or sham burn groups according to a random digits table, and the burn group was subdivided into groups of post-burn at 1 h, 3 h, 6 h, and 12 h time points (n = 8 for each group). Control non-burn sham animals were also included (n = 8). Animals were intraperitoneally injected with 1 g/L pentobarbital sodium (30 mg/kg). Under anesthesia, the hair on the back of the rat was shaved, and the nude skin was scalded with a 97°C water bath for 18 seconds, sufficient to cause a 3rd degree burn encompassing 30% total body surface area (TBSA), which was confirmed post-mortem. The sham burn group had the same procedure, except the temperature of the water was 37°C. After immersion, each animal was placed in an individual cage and allowed free access to food and water. The burn rats were resuscitated with an intraperitoneal (i.p.) injection of a volume of Ringer’s lactate following the Parkland formula (4 ml/kg/1%TBSA). The total calculated volume of fluid for the first 24 h was delivered in three injections at 30 min, 4 h and 8 h post-burn (1/4 total volume for each).

### Assay of Cardiac Function in vivo

For measurement of cardiac function in vivo, the neck region of the rats was prepared for surgery under anesthesia and analgesia (pentobarbital 30 mg/kg i.p.). The right carotid artery was exposed, and a polyethylene catheter (i.d. 0.5 mm) filled with isotonic saline containing 25 U/L heparin was inserted through the right common carotid artery into the aorta, and the other end linked to a pressure transducer of the Multi-Channel Physiological Signal Collecting and Processing System (RM6420 model, Chengdu Instruments Factory, China). Five minutes later, mean arterial pressure (MAP) were measured by the RM6420 software of the system. The catheter was then inserted further into the left ventricle to record and analyze left ventricular systolic pressure (LVSP), left ventricular end-diastolic pressure (LVEDP) and maximal rate of the rise/drop of left ventricular pressure (± dp/dt max), which reflected left ventricular systolic and diastolic function. The parameters were measured immediately after sham burn in sham group, or measured at the indicated time points in the burn group. After the measurements, rats were killed by cervical neck dislocation, and the hearts were harvested.

### Perfusion and Functional Assay of Isolated Rat Heart

The hearts were removed rapidly from rats under anesthesia (pentobarbital 30 mg/kg i.p.) and mounted on a Langendorff apparatus. The hearts were perfused with Krebs-Henseleit (K-H) buffer containing (in mM) 118.5 NaCl, 4.7 KCl, 1.2 MgSO_4_, 1.8 CaCl_2_, 24.8 NaHCO_3_, 1.2 KH_2_PO_4_, and 10 glucose, which was heated to 37°C and gassed with 95% O_2_/5% CO_2_. A latex balloon connected to a pressure transducer was inserted into the left ventricle through the left atrium. The left ventricular pressure was continuously recorded with the Multi-Channel Physiological Signal Collecting and Processing System (RM6420 model, Chengdu Instruments Factory, China). All hearts were allowed to stabilize for at least 20 min. The hearts from rats 6 h post-burn were then perfused with K-H buffer with 0.1 µM rapamycin (an activator of autophagy, from LKT Laboratories, USA), 1 mM 3-MA (an inhibitor of autophagy, from Sigma, USA), 5.7 µM enalaprilat (an angiotensin converting enzyme inhibitor [ACEI], Changzhou Pharmaceutical Factory, China), 0.1 mM losartan (an AT1 receptor blocker, LKT Laboratories, USA), or 5 µM DPI (a reactive oxygen species [ROS] production inhibitor, Sigma, USA) for 30 min before determining cardiac function again. Continuous K-H buffer perfusion was added to isolated hearts from the 6 h post-burn group as a control.

### Immunolabeling and Fluorescence Microscopy

The apex of the heart (about 100 mg in weight) was separated and quickly frozen in liquid nitrogen. The tissue blocks were then prepared as frozen sections (5 µm thick) which were fixed for 10 minutes with 4% paraformaldehyde and then exposed for 10 minutes in 1% BSA, followed by incubation with the corresponding antibodies in double or triple staining procedures. The primary antibodies used included rabbit polyclonal anti-ubiquitin (Abcam, USA), mouse monoclonal anti-actin (Abcam, USA), and mouse monoclonal anti-C5b9 (Santa Cruz, USA). The secondary detection system was biotinylated anti-mouse or anti-rabbit IgG (Byotime, China) conjugated with Cy-3 or fluorescein isothiocyanate (FITC). In situ labeling of fragmented DNA (TUNEL) was performed in the sections using a commercially available kit (Beyontime, China). Nuclei were stained with DAPI (Sigma, USA). Omission of primary antibodies served as a negative control. The samples were examined with a digital fluorescence microscope (DM6000B, Leica, Germany).

### Quantification of Ubiquitin-, TUNEL-, and C5b9-positive Myocytes

From each tissue block, 3 sections 5 µm thick, cut at a distance of 100 µm, were stained and quantitatively evaluated. Counterstaining for identification of myocytes was done with anti-actin combined with a secondary fluorescent antibody. Ubiquitin-positive cardiomyocytes were counted for the entire section. The number of myocyte nuclei per 5 randomly chosen fields of vision (×400) was counted and calculated per mm^2^. From these data and the area of the tissue section, the total number of myocytes was determined, and ubiquitin-positive cells were expressed as a percentage of the total number of cardiomyocyte nuclei. The same counting procedure was performed for TUNEL and C5b9 labeling.

### Immunoblotting

Protein was extracted in RIPA buffer from the myocardium tissue blocks and stored at -80°C. For each lane, 30 µg protein was loaded and separated on a 10% sodium dodecyl sulfate-polyacrylamide gels and transferred onto nitrocellulose membranes. After the blocking procedure, the membrane was immunoblotted with the primary antibodies: rabbit polyclonal anti-LC3b (Cell Signaling, USA) and/or rabbit polyclonal anti-beclin 1 (Cell Signaling, USA) at 4°C overnight. After washing, the membrane was incubated with secondary antibodies (Santa Cruz, USA) for 1 hour at room temperature. The immunocomplexes were visualized with an enhanced chemiluminescence detection kit (Amersham Pharmacia, USA) and the densities of the bands were quantified with QuantityOne software (Bio-Rad, USA). GAPDH (rabbit polyclonal anti-GAPDH from Sigma, USA) was also probed and visualized as a loading control.

### Statistics

All data are presented as mean ± SEM. SPSS 13.0 was used for statistical analysis and significance evaluated by one-way ANOVA followed by the Student-Newman-Keuls (SNK) post-hoc test. Differences were considered statistically significant when P < 0.05.
